# Multiple Inherited Thrombophilic Gene Polymorphisms in Spontaneous Abortions in Turkish Population

**Published:** 2015

**Authors:** Sinem Yalcintepe, Ozturk Ozdemir, Servet Ozden Hacivelioglu, Cisem Akurut, Evrim Koc, Ahmet Uludag, Emine Cosar, Fatma Silan

**Affiliations:** 1 *Department of Medical Genetics, Faculty of Medicine, Canakkale Onsekiz Mart University, Canakkale, Turkey.*; 2 *Genetics Diagnosis Center, Numune Training and Education Hospital, Adana, Turkey.*; 3 *Department of Medical Genetics, Faculty of Medicine, Cumhuriyet University, Sivas, Turkey.*; 4 *Department of Gynecology and Obstetrics, School of Medicine, Canakkale Onsekiz Mart University, Canakkale, Turkey.*

**Keywords:** Spontaneous abortion, thrombophilia, polymorphism, fetus

## Abstract

The aim of this study was to investigate the possible role of multiple inherited thrombophilic gene variations in women with unexplained spontaneous abortions**.** For this purpose, the Factor V Leiden (*FVL*) (rs6025), Prothrombin G20210A (rs1799963), *MTHFR* C677T (rs1801133), *PAI-1* 4G/5G (rs1799889), *ACE I/D* (rs1799752), *eNOS* E298D (rs1799983), and *Apo E* E2/E3/E4 (rs429358) polymorphisms were genotyped and correlated in spontaneously aborted fetal materials, their mothers and fertile women. Twenty three abortion materials, 22 women with ≥1 unexplained fetal loss, and 22 control subjects with at least two healthy term infants as a control group were studied. Target SNPs for each gene were analyzed by real time-PCR technique after genomic DNA isolation from maternal blood-EDTA, control group blood-EDTA and spontaneously aborted fetal tissues. Some cases had a single thrombophilic polymorphism, but the rest of the patients and fetal materials had combined thrombophilic polymorphisms. The *PAI-1* 4G/5G+4G/4G (P= 0.0017), 4G/4G (P= 0.0253), *eNOS* 894GT+894TT (P=0.0011) genotypes and T allele (P=0.0185), *Apo E* E3/E4+E3/E2+E2/E4 (P<0.0001) genotypes, E2 (P<0.0001) and E4 (P<0.0001) alleles were higher in spontaneously aborted fetal materials when compared to their mothers and control group. The Factor V Leiden rs6025, Prothrombin G20210A, *MTHFR* C677T, *ACE I/D* genotypes were different for each group but not statistically significant due to relatively small size of the samples (P>0.05). Our results indicated that combined thrombophilic gene variations may be associated with increased risk for spontaneous abortions and results need to be confirmed by larger sample size.

Spontaneous abortion is defined as a clinically recognized pregnancy loss before the gestation week of twenty. The World Health Organization (WHO) defines it as expulsion or extraction of an embryo or fetus weighting 500 g or less (1). A normal pregnancy is dependent on sufficient placental circulation and fetal vasculature. Abnormalities of placental vasculature may cause several gestational complications like pregnancy loss, intrauterine fetal death, intrauterine growth restriction and preeclampsia (2, 3). The risk of pregnancy loss is enhanced by a variety of etiological factors, including chromosomal abnormalities, uterine abnormalities, endocrinolo-gical defects, infections and environmental factors. However, in up to 50% of cases the exact underlying pathophysiological mechanisms remain undetermined.

Recurrent pregnancy loss (RPL) is an important obstetric complication which is defined as two or more miscarriages of under 20 weeks of gestation (4). Thrombophilias are inherited or acquired conditions that predispose an individual to thromboembolism (5). Inherited thrombophilia has been known as a cause of RPL. Various studies in the recent years have examined the incidence of specific thrombophilic polymorphisms in women with RPL. Some studies have reported an association between thrombophilic gene mutations and RPL (6, 7), whereas others have shown the lack of any association (8, 9).

In this study, we aimed to compare the thrombophilic polymorphisms in Factor V Leiden (*FVL*) rs6025, Prothrombin G20210A (rs1799963), *MTHFR* C677T (rs1801133), *PAI-1* 4G/5G (rs1799889), *ACE I/D* (rs1799752), *eNOS* E298D (rs1799983), and *Apo E* E2/E3/E4 (rs429358) in spontaneously aborted fetal materials with their mothers and fertile cases. This is the first study about thrombophilia polymorphisms in spontaneously aborted fetal materials.

## Materials and methods

**Patients and clinical diagnosis**

Twenty-three abortion materials, their twenty- two mothers (one of the mothers had two abortions) and twenty-two fertile women as control group were included in this study. Abortion materials were obtained from only spontaneous abortions. Maternally contaminated abortion materials (twenty-seven materials) were excluded from the study (fifty abortion materials were taken for this study). Maternal contamination was detected with correlation of fetus and mother STR markers. Control group women had no spontaneous abortions and they had two or more children. All participants were included from medical genetics clinic referred from Gynecology and Obstetry clinic in Canakkale On Sekiz Mart University Hospital.

Written informed consent forms were obtained from all participants and the study was approved by the ethics committee of Canakkale On Sekiz Mart University.

**Genotyping**

All samples were genotyped for Factor V Leiden (*FVL*) (rs6025), Prothrombin G20210A (rs1799963), *MTHFR* C677T (rs1801133), *PAI-1* 4G/5G (rs1799889), *ACE I/D* (rs1799752), *eNOS* E298D (rs1799983), and *Apo E* E2/E3/E4 (rs429358) polymorphisms.

**Fig. 1:**
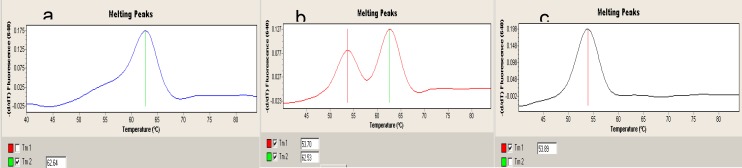
Shows the Real-time-PCR profiles of *MTHFR* C677T genotypes that evaluated for the studied groups. **a:** Homozygous CC genotype (Tm: 63 ^0^C). **b:** Heterozygous CT genotype (Tm: 54.5 ^0^C and 63 ^0^C). **c:** Homozygous TT genotype (Tm: 54.5 ^0^C

Total genomic DNA was isolated from either 10-15 mg of abortion material tissue by the QIAGEN QIAamp DNA Mini Kit or peripheral blood samples containing EDTA from the mothers and control cases by the QIAGEN QIAamp DNA Blood Mini Kit. The polymorphic alleles were ampliﬁed by real- time polymerase chain reaction (PCR) technique in Light Cycler 2.0 (Roche, Switzerland) using Light Mix kits (TIB MOBIOL, Germany). Brieﬂy, Light Cycler, Roche Fast Start Master mix, master mix (water, PCR- grade, Mg^+2^, stock solution, Primer mix and HybProbe mix) and DNA template were used for real-time ampliﬁcation. The multiple PCR consisted of a denaturation step of 10 min at 95 ^o^C, followed by 45 cycles of 5 s at 95 ^o^C,10 s at 60 ^o^C, and 15 s at 72 ^o^C, and a melting step of 20 s at 95 ^o^C, 20 s at 40 ^o^C, a continuous mode at 85 ^o^C, a cooling step of 30 s at 40 ^o^C for all four regions. A software program (Light Cycler 2.0, Roche, Switzerland) was used for the detection of the mutated and normal genotype proﬁles of the target gene in the groups included in the study. The wild, heterozygous and homozygous profiles were analysed in channel 640 with melting curve analysis (Figure 1).

***Statistical analysis***

Statistical analysis has been done with medcalc statistical program. Chi-square test was used to analyze the differences between the abortion materials, mothers and fertile women as control group. Genotype percentages and allele frequencies were compared between aborted materials group-mothers group and aborted materials group-control group. Odds ratio (OR) and P-values were used to estimate the risk for the polymorphisms in the groups.

## Results

Peripheral blood-EDTA samples from mothers and control cases (fertile women) and tissue samples from aborted materials were examined for genotyping in the current study. Mean age of the cases and the weeks of abortions are provided. The mean age of the mothers were 29.6 (21-40) years and the mean age of fertile women were 37.2 (30-46) years. The mean abortion week was 9.4 (5-18) weeks, twenty abortions were in first trimester, three abortions were in second trimester. There was no other diseases in the groups.

The fetus, mothers and control groups in this study were designed to determine the association between point mutations in the factor V Leiden (*FVL*), protrombin G20210A,  methylenetetra-hydrofolate reductase (*MTHFR*) C677T, plasminogen activator inhibitor-1 (*PAI-1*) 4G/5G, Angiotensin-converting enzyme I/D (*ACE I/D*), endothelial nitric oxide synthase (*eNOS*) E298D, apolipoprotein E (*Apo E*) E2/E3/E4 and spontaneous abortions. By real time PCR technique, we evaluated these polymorphisms in the study groups (23 abortion materials, 22 mothers, 22 fertile women), and the results were compared between the groups. The estimate risk was examined by odds ratio.

10 aborted materials and 6 mother results for *PAI *4G/5G, 3 mother results for *eNOS* E298D, 3 aborted materials and 2 mother results for *Apo E* polymorphisms, 2 mother results for *ACE I/D* polymorphism were missed because of unclear PCR results. Genotype frequencies and statistical results are presented in table 1. The statistical analysis was done in two different ways. All genotype and allele frequencies were compared between the groups. In statistical analysis, we used four different comparings:1. Percentages of wild genotype versus heterozyg-ous mutant+homozygous mutant genotypes.2. Percentages of wild genotype +heterozygous mutant genotype versus homozy- gous mutant genotypes. 3. Percentages of wild genotype versus homozygous mutant genotypes. 4. Allele frequencies.

**Tables 1:** The genotypes and allelle frequency of target *PAI*-1 4G/5G, *eNOS* E298D gene polymorphisms in  aborted materials and their mothers in the current limited results

***Genes/SNPs***	***Groups***				
	*Aborted* *Materials (n:13)*	*Mothers (n:16)*			
*PAI-1 4G/5G*	*n/%*	*n/%*			
*5G/5G*	*4/30.8*	*2/12.5*			
*5G/4G*	*5/38.4*	*9/56.2*			
*4G/4G*	*4/30.8*	*5/31.3*	**Statistical Analysis**		
**Allele Frequency**			**P value**	**Odds ratio**	**Cl (95 %)**
5G	13(0.500)	13(0.406)	<0.0017^a^	3.2572	1.5580-6.8098
4G	13(0.500)	19(0.594)			
eNOS E298D (G894T)	*AB(n:23)*	*M (n:19)*			
GG	16/69.6	9/47.4			
GT	7/30.4	9/47.4			
TT	-	1/5.2			
**Allele Frequency**					
G	39(0.848)	27(0.711)			
T	7(0.152)	11(0.289)*	0.0185*	2.3146	1.1511-4.6539

**AB:** Aborted Materials; **M:**Mothers

* **: **Significant,

a Pearson Chi-Square;

**Tables 2:** The genotypes and allelle frequency of target *PAI*-1 4G/5G, *eNOS* E298D gene polymorphisms in  aborted materials and fertile couples (controls) in the presented results

**Genes/ SNPs**	**Groups**				
	Aborted Materials (n:13)	Fertile Women (n:22)			
PAI-1 4G/5G	n/%	n/%			
5G/5G	4/30.8	9/41			
5G/4G	5/38.4	10/45.5			
4G/4G	4/30.8	3/13.5	**Statistical Analysis**		
**Allele Frequency**			**P value**	**Odds ratio**	**Cl (95 %)**
5G	13(0.500)	28(0.636)	0.0028^a^	3.0067	1.4626-6.1807
4G	13(0.500)*	16(0.364)			
**eNOS **E298D (G894T)					
GG	16/69.6	14/47.4			
GT	7/30.4	7/47.4			
TT	-	1/5.2			
**Allele Frequency**					
G	39(0.848)	35(0.795)			
T	7(0.152)	11(0.205)*	0.3535^a^	0.7059	0.3382-1.4733

* ***:*** *Significant,*

a *Pearson Chi-Square*

For PAI-1 4G/5G polymorphism, 4G/5G+4G/ 4G genotypes percentages (P=0.0017) and 4G/4G genotype (p=0.0253) were higher in aborted materials comparing with their mothers (Table 1). 4G/4G genotype was also higher in aborted materials comparing with the control group (P=0.0028) (Table 2). For eNOS G894T polymor- phism, GT+TT genotype percentages (P=0.0011) and T allele frequency (P= 0.0185) were higher in aborted materials comparing with their mothers (Table 1). For *Apo* E2/E3/E4 polymorphism, the sum of the polymorphisms except E3/E3 (P<0.0001, P=0.0019), E2 allele frequency (P<0.0001, P= 0.0041) and E4 allele frequency (P<0.0001 for both) were higher in aborted materials comparing with their mothers and with control group, respectively (Tables 3-4). *FVL*, Prothrombin G20210A, *MTHFR* C677T, *ACE I/D* polymorphisms were not significant when comparing aborted materials with both their mothers and control group (P> 0.005) (Table 5).

**Tables 3:** Shows the genotypes and allelle frequency of ApoE E2/E3/E4 polymorphisms in  aborted materials and their mothers in the current limited results

**Genes/SNPs**	**Groups**					
	Aborted Materials (n:21)		Mothers (n:20)			
ApoE E2/E3/E4	n/%		n/%			
E3/E3	7/33.3		14/70			
E3/E2	11./52.4		5/25			
E3/E4	2/9.5		1/5			
E2/E4	1/4.8		-			
**Allele Frequency**				**Statistical Analysis**		
E3	27/0.643	34/0.850		**P value**	**Odds ratio**	**Cl (95 %)**
E2	12/0.286*	5/0.125				
E4	3/0.071*	1/0.025		**<0.0001** a	3.7188	2.3288-5.9384

* ***:*** *Significant,*

a *Pearson Chi-Square*

**Tables 4:** The genotypes and allelle frequency of ApoE E2/E3/E4 polymorphisms in  aborted materials and fertile couples in the presented results

**Genes/SNPs**	**Groups**				
	Aborted Materials (n:21)	Fertile Women (n:22)			
ApoE E2/E3/E4	n/%	n/%			
E3/E3	7/33.3	12/54.5			
E3/E2	11./52.4	4/18			
E3/E4	2/9.5	5/23			
E2/E4	1/4.8	1/4.5			
**Allele Frequency**			**Statistical Analysis**		
E3	27/0.643	33/0.750	**P value**	**Odds ratio**	**Cl (95 %)**
E2	12/0.286*	5/0.114			
E4	3/0.071*	3/0.136	**<0.0001** a	5.8594	3.0172-11.3789

* ***:*** *Significant,*

a *Pearson Chi-Square*

Three abortion materials had both *MTHFR* C677T and *eNOS* E298D mutations, five abortion materials had both *MTHFR* C677T and *ACE I/D* mutations, eight abortion materials had both *PAI-1 *4G/5G and *ACE I/D* mutations, three abortion materials had both *eNOS* E298D and *Apo E* mutations, eight abortion materials had both *ACE I/D* and *eNOS* E298D mutations.

## Discussion

Thrombophilia is one of the coagulation disorders which predisposes individuals to develop thrombosis (10). It includes acquired thrombophilia such as antiphospholipid antibody syndrome, activated protein C resistance, deficiencies in antithrombin III (ATIII), proteins C, S, and heritable causes such as factor V Leiden, Prothrombin (Factor II) G20210A, *MTHFR* C677T, *PAI-1* 4G/5G, * ACE I/D*, * eNOS* E298D, *Apo E* E2/E3/E4. These thrombophilic factors may have an important role in influencing the pregnancy outcome. This is the first study to report the relationship between thrombophilic factors polymorphisms and pregnancy loss in spon-taneously aborted fetal materials and their mothers.

**Tables 5:** The statistically significance of the genotypes and allelle frequency of target genes for *FVL*, prothrombin G20210A, *MTHFR* C677T, *ACE* I/D SNPs in  aborted materials, their mothers and fertile couples in the presented results**.**

***Genes/SNPs***	***GROUPS***			***Statistical Analysis***		
	***Aborted*** ***Materials*** ***(n:23)***	***Mothers*** ***(n:22)***	***Fertile Controls*** ***(n:22)***	***P value***	***OR***	***CI(95%)***
*FVL*						
*GG* *GA* *AA*	*21/95* *2/5* *-*	*21/95.5* *1/4.5* *-*	*22/100* *-* *-*			
*Allele Frequency*						
*G*	*44/0.960*	*43/0.980*	*44/1.000*	*0.0989*	*11.5759*	*0.6315 -*
*A*	*2/0.040* *	*1/0.040* *	*0/0.000*			*212.2057*
*Prothrombin G20210A* *GG* *GA* *AA* *Allele Frequency* *G* *A*	*23/100* *-* *-* *46/1.000* *0/0.000*	*22/100* *-* *-* *44/1.000 0/0.000*	*22/100* *-* *-* *44/1.000* *0/0.000*	*1.0000*	*1.0000*	*0.0196 - 50.8937*
*MTHFR C677T * *CC* *CT* *TT* *Allele Frequency* *C* *T*	*11/48* *11/48* *1/4* *33/0.720* *13/0.280*	*10/45.5* *11/50* *1/4.5* *31/0.700* *13/0.300*	*11/50* *8/36.4* *3/13.6* *30/0.680* *14/0.320*	*0.7773*	*1.0833*	*0.6222 -1.8863*
*ACE I/D* *I/I* *I/D* *D/D* *Allele Frequency* *I*	*5/21.8* *6/26* *12/52.2* *16/0.350 30/0.650* *	*4/20* *7/35* *9/45* *15/0.380* *25/0.620* *	*5/22.8* *9/40.9* *8/36.3* *19/0.430* *25/0.570*	*0.8634*	*1.0611*	*0.5403- *
*D*						*2.0836*

* **: **Significant; 95% CI for FVL;MTHFR C677T; and ACE I/D

The placenta has double blood supply, one maternal and one fetal. The maternal blood, originating from uterine spiral arteries, circulates in the intervillous space, comes into contact with the syncytiotrophoblast and drains back through decidual veins. The fetal blood supply within the placenta starts from the umbilical arteries. Blood circulates in the fetal villi and drains back via the umbilical vein. A hypercoagulable state within the fetal circulation could lead to fetal stem vessel thrombosis, placental infarction in the distribution of fetal vessels and a miscarriage. In a study, the presence of *FVL* mutation in fetuses has been reported to be associated with histologically proven placental infarction (11). In our study, *FVL* genotype was not significant in fetal carriage comparing with their mothers for spontaneous abortions. In the literature, many studies have been reported about the relationship of FVL and other trombophilic factors about usually recurrent pregnancy loss and women genotypes. Sottilotta et. al. reported that FVL and the FII G20210A mutations were related with unexplained stillbirth, but not with recurrent pregnancy loss (12). In another study, FVL and MTHFR polymorphisms were found as not associated with recurrent pregnancy loss or pre- eclampsia (13).Generally, studies belong to different countries and races, so that polymorphism results may differ. For example, a study from China reported that there was a significant association between MTHFR C677T and unexplained recurrent pregnancy loss (14). A study planned with couples, reported that MTHFR C677T polymorphism is a risk factor for RPL (15). Zetterberg et.al reported that combined MTHFR C677T and A1298C mutations were higher in spontaneously aborted embryos comparing with adult controls (16). We did not detect any association between MTHFR C677T polymorphism and spontaneous abortion in this study.

Differently, in a study fetal karyotypes were analyzed following miscarriages and 41.4% had chromosomal abnormalities. After analyzing their parents’ karyotypes they reported that there is a relationship between chromosome abnormalities and spontaneous abortion (17). In contrast usual studies, Udry et.al. investigated the relationship between paternal FVL and prothrombin G20210A genotypes and recurrent pregnancy loss. It was reported that paternal FVL mutation was associated with RPL (18). These results partly remind our study with including fetal and paternal genetic results to their studies. It is clear that it is important to include not only maternal results to pregnancy loss studies but also paternal and fetal results. A fetus takes the genes both maternal and paternal, so that a pregnancy loss can not be only related with women chromosome abnormalities or genotypes.

With an overview to this study, *FVL*, Prothrombin G20210A, MTHFR C677T, ACE I/D polymorphisms were not different in spontaneously aborted fetal materials and both mothers and contol group. Only PAI-1 4G/5G, eNOS E298D and Apo E E2/E3/E4 polymorphisms had higher prevalence in fetal materials comparing with their mothers and fertile women. Studies about miscarriages usually researched recurrent pregnancy losses and women genotypes for thrombophilic factors. Subrt et. al. reported that PAI-1 (-675) 4G/4G homozygous genotype was associated with increased risk of RPL independently from the antiphospholipid antibodies (19). In a different study, it was reported that ACE I/D, PAI-1 4G/5G and NOS3 4a/4b were not associated with first trimester recurrent miscarriage (20). Agarwal et.al. reported that there was no association between *Apo E *gene polymorphisms and RPL (21). In our results Apo E2 and E4 alleles and genotypes with these alleles were significant comparing with E3 allele and E3/E3 genotype between fetus and mother groups. Fetal Apo E genotype is found as associated with spontaneous abortion.

In conclusion, spontaneous abortion risk is related with PAI-1 4G/5G, eNOS E298D and Apo E E2/E3/E4 polymorphisms for fetal genotypes. If fetal genotypes are effective comparing with mother genotypes, this means father genotypes are also effective for pregnancy losses. Our results indicated that combined thrombophilic gene mutations may be associated with increased risk for spontaneous abortions. There is a need for larger studies to explore the effect of thrombophilic factors in family members for pregnancy losses.
